# Carbon storage in rare ecosystems relative to their encroaching forests in western Lower Michigan

**DOI:** 10.1371/journal.pone.0305394

**Published:** 2024-06-17

**Authors:** M. Megan Woller-Skar, Alexandra Locher, Ellen M. Audia

**Affiliations:** 1 Department of Biology, Grand Valley State University, Allendale, MI, United States of America; 2 Cooperative Wildlife Research Laboratory, Southern Illinois University Carbondale, Carbondale, IL, United States of America; Western Carolina University, UNITED STATES

## Abstract

Rising atmospheric carbon dioxide levels are impacting global temperatures, ecological systems, and human societies. Natural carbon sequestration through the conservation of soil and native ecosystems may slow or reduce the amount of CO_2_ in the atmosphere, and thus slow or mitigate the rate of global warming. Most of the research investigating carbon sequestration in natural systems occurs in forested ecosystems, however rare ecosystems such as coastal plain marshes and wet-mesic sand prairie collectively may serve as significant carbon sinks. Our objectives were to measure and assess the importance of carbon sequestration in three rare ecosystems (oak-pine barrens, coastal plain marsh, and wet-mesic sand prairie) in western Lower Michigan. We measured carbon in standing vegetation, dead organic matter, and soils within each ecosystem and adjacent encroaching forested areas. Driven by tree carbon, total carbon stocks in encroaching areas were greater than in intact rare ecosystems. Soil organic carbon was greater in all intact ecosystems, though only significantly so in coastal plain marsh. Principal components analysis explained 72% of the variation and revealed differences between intact ecosystems and their encroaching areas. Linear models using the ratio of red to green light reflectance successfully predicted SOC in intact coastal plain marsh and wet-mesic sand prairie. Our results infer the importance of these rare ecosystems in sequestering carbon in soils and support the need to establish federal or state management practices for the conservation of these systems.

## Introduction

The amount of carbon dioxide (CO_2_) in the earth’s atmosphere is steadily rising. Researchers report that during pre-industrial times (~250 years ago), for every 1 million air molecules, 280 of them were CO_2_ (i.e., 280 parts per million (ppm) [1]. Today, CO_2_ levels have reached over 400 ppm [1]. The sources of this carbon are varied but fossil fuel combustion is known to be a major contributor to increasing, potent CO_2_ [2]. Carbon sequestration may slow or reduce the amount of CO_2_ in the atmosphere, and thus slow or mitigate the rate of global warming [3].

Natural carbon sequestration involves processes by which atmospheric carbon is removed and stored over time [4], which may occur through the conservation of soil and native ecosystems. Globally, forests serve as carbon sinks and can potentially store about 50% of atmospheric carbon [5]. Wetlands may sequester more than 40% of atmospheric carbon [6] and grasslands may store a significant amount of biological carbon (0–8 tC/ha) as well [7, 8]. Preserving carbon sequestration in these terrestrial ecosystems is achieved through conservation of aboveground and belowground biomass.

Much of the research investigating carbon sequestration in natural systems occurs in forested ecosystems, as forests occupy nearly 30% of the Earth’s land surface [9]. However, rare ecosystems may also serve an important role in storing carbon. Although they are often small and scattered within landscapes, rare ecosystems may collectively represent significant carbon sinks [10], which can help offset global carbon imbalances [11]. Except for a few studies regarding freshwater coastal marshes in Illinois [12–14] and Ohio [15–17], little is known about the value of these systems for storing carbon; however, their potential is high because they have largely been intact since glacial times [18].

Oak-pine (Pinus spp.) barrens (OPB), coastal plain marshes (CPM), and wet-mesic sand prairies (WMP) are recognized as rare ecosystems in the State of Michigan by the Michigan Natural Features Inventory (MNFI, 19, Table 1). Furthermore, MNFI identified these ecosystems as being at high to moderate risk of extinction from the state of Michigan and globally (19, Table 1) due to changes in natural disturbances, land use practices, or encroachment of woody vegetation. Therefore, there is tremendous value in investigating the role of these rare systems in storing carbon and their potential importance in helping mitigate the effects of climate change. Such knowledge could guide management efforts that prioritize the conservation of these rare ecosystems.

**Table 1:** Rankings and criteria from the Michigan Natural Features Inventory (MNFI) for the three rare ecosystems included in this study. For more detailed information see [19].

Ecosystem	Rank	Code	MNFI Ranking Criteria
Coastal Plain Marsh	Global Rank	G2	High risk of extinction
	State Rank	S2	Very vulnerable to extirpation from Michigan
Oak-Pine Barrens	Global Rank	G3	Moderate risk of extinction
	State Rank	S2	Very vulnerable to extirpation from Michigan
Wet-Mesic Sand Prairie	Global Rank	G2/G3	Moderate to high risk of extinction
	State Rank	S2	Very vulnerable to extirpation from Michigan

The goal of this project was to measure carbon stocks in three rare ecosystems (OPB, CPM, and WMP) in western Lower Michigan. Specifically, we aimed to 1) quantify carbon in aboveground tree biomass, herbaceous vegetation, dead organic matter, and soil (SOC) in OPB, CPM, and WMP, as well as adjacent encroaching forest areas; 2) compare carbon stocks in these rare ecosystems and encroaching areas, and 3) develop robust observations and models to explain total carbon and SOC in these ecosystems using high-resolution spectral reflectance.

### Study area

**Fig 1:**
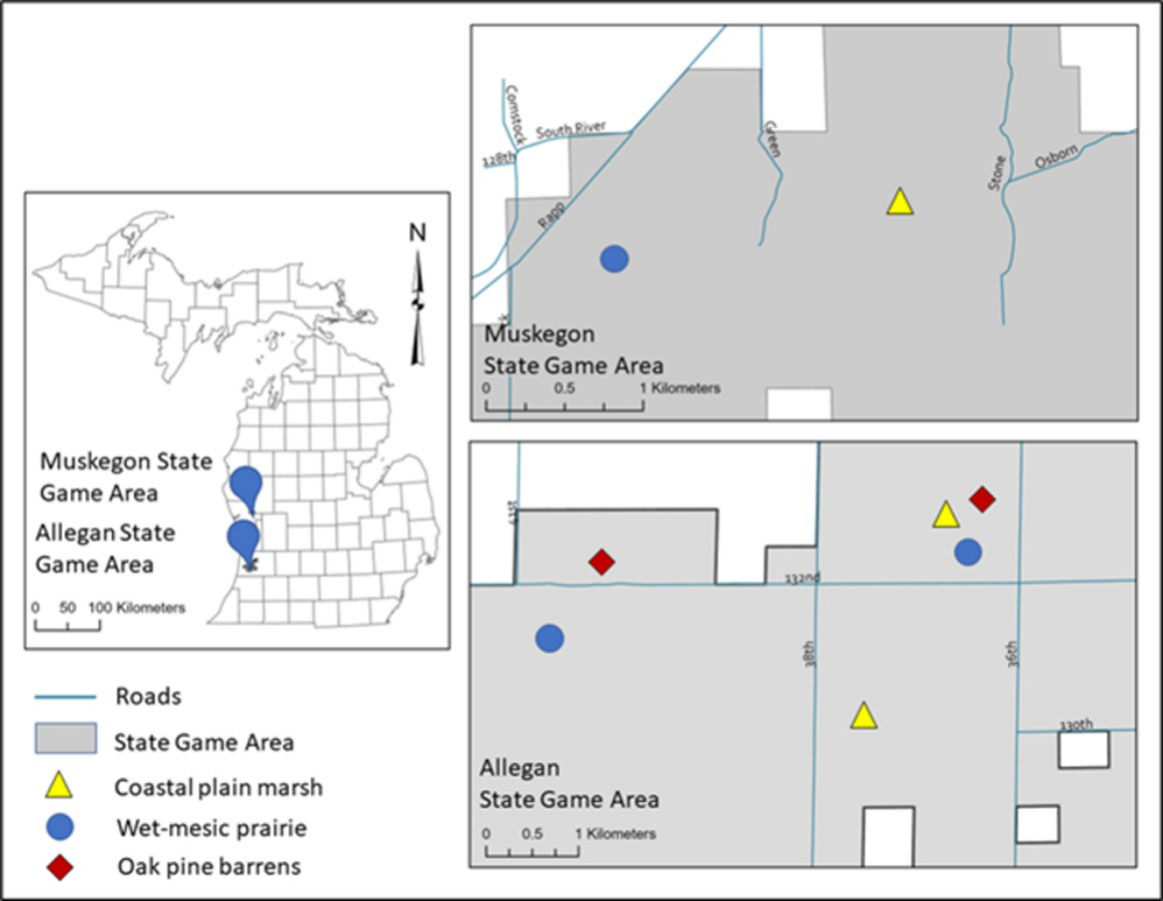
Location of rare ecosystems within Allegan State Game Area (bottom) and Muskegon 87 State Game Area (top). County boundary basemap (left) and state game area boundaries (right) were accessed from Michigan GIS Open Data (gis-michigan.opendata.arcgis.com; search “Counties (v17a)” and “State Forest campgrounds and forest boundaries”).

We conducted fieldwork and directly measured and modeled carbon within Allegan State Game Area (ASGA) and validated models at the Muskegon State Game Area (MSGA). Allegan State Game Area is in Allegan County, Michigan, USA, and MSGA is approximately 74 km away in Muskegon and Newaygo counties, Michigan, USA (Fig 1). These state game areas are managed by the Michigan Department of Natural Resources for wildlife, particularly game species, including white-tailed deer (*Odocoileus virginianus*), eastern wild turkey (*Meleagris gallopavo*), and waterfowl. The ASGA supports rare ecosystems including OPB, CPM, and WMP. The MSGA supports CPM and WMP.

Oak-pine barrens are considered imperiled communities in Michigan, with fewer than 20 occurrences in the state and < 1% of their historical extent remaining [19]. Oak-pine barrens were historically represented by a shifting dominance of oak-pine and herbaceous grasses and forbs, depending on fire regimes and drought. Fires intense enough to kill canopy trees and create openings created barrens that are now maintained, in part, by low-intensity surface fires [19]. The vegetation composition in CPM resembles that of the Atlantic and Gulf coastal plains. Fluctuating water levels are the dominant natural process maintaining the structure, species composition, and functions of these systems [19]. Coastal plain marshes are also globally imperiled [19]. Wet-mesic sand prairies, characterized by lowland grassland communities occurring on sandy outwash plains, are also imperiled, with fewer than 10 remaining in Michigan [19]. They originated on glacial lake plains and have been sustained by fluctuating levels of drought and high-water tables that help prevent woody encroachment.

These rare ecosystems at ASGA and MSGA were originally brought to our attention by MNFI in 2015. Michigan Natural Features Inventory also determined that the forest vegetation surrounding these rare ecosystems was encroaching, posing a severe threat to the persistence of OPB, CPM, and WMP. For this study, we identified encroaching areas as boundaries between rare systems (OPB, CPM, and WMP) and neighboring woodlands. Encroaching forests will hereafter be referred to as “encroaching” and rare ecosystems will hereafter be referred to as “intact”.

## Methods

### Abiotic data

To characterize abiotic environmental variables, we measured soil temperature, soil pH, air temperature, relative humidity, and wind speed and direction at each site using a soil multiprobe and a Kestrel 3000 weather meter.

### Carbon storage

There are two frequently used methods for measuring carbon sequestration in terrestrial systems. The first is to collect biomass, dry it in ovens, and convert the weight of the resulting oven-dried biomass to units of carbon [20]. A second method is to monitor vegetation from high-resolution satellite imagery and compute indices based on spectral reflectance, which may indicate levels of photosynthetic activity and biomass [21]. Combining on-the-ground and remotely sensed methods allows researchers to develop models to predict amounts of carbon in areas exhibiting similar characteristics within the landscape [22].

**Fig 2:**
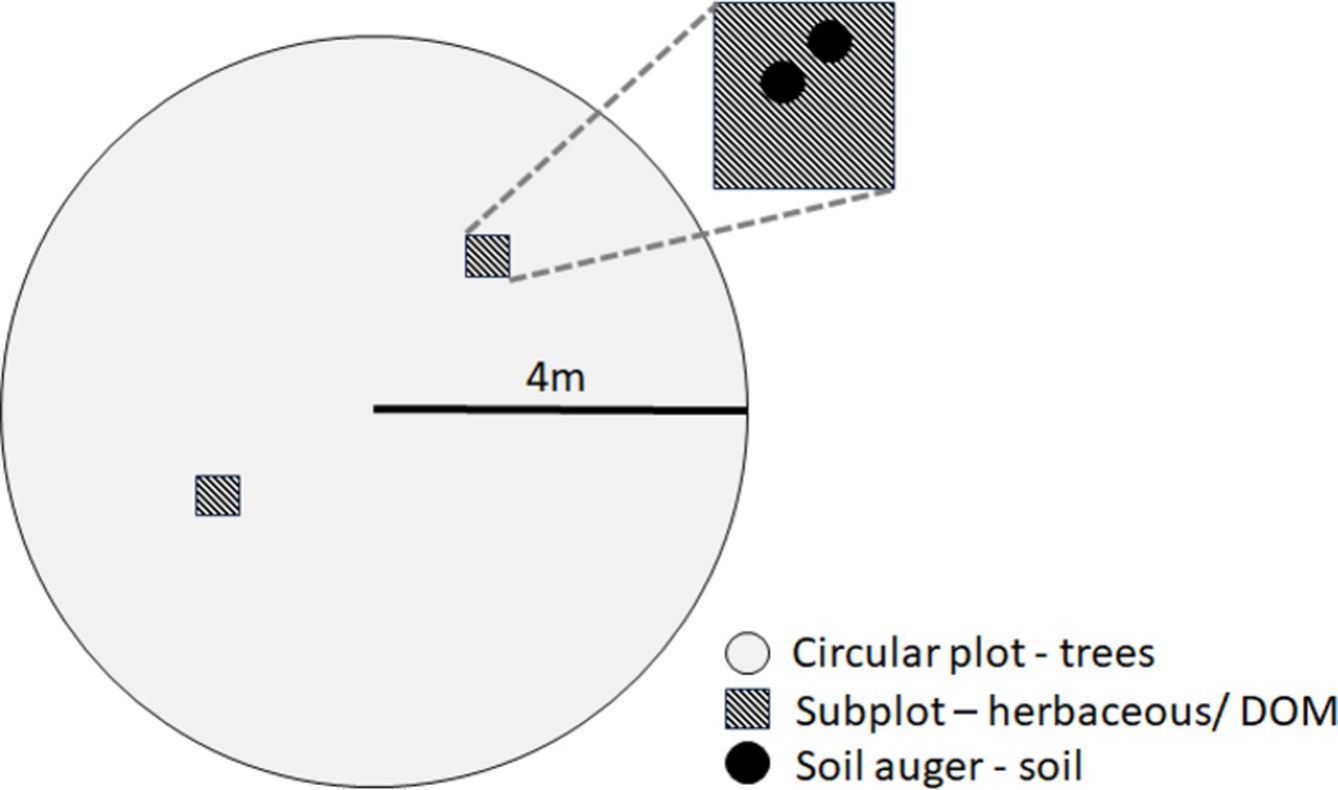
Schematic of sampling for estimation of carbon stocks. Trees were sampled in circular plots with a 4 m radius (n = 20 for intact, n = 20 for encroaching, for each ecosystem). Herbaceous and dead organic matter (DOM) were collected from two 0.25 m^2^ subplots (summed per circular plot). After herbaceous and DOM removed from subplots, soil was collected using a soil auger from 2 locations in each subplot, or 4 cores per circular plot (total of 80 cores per intact, n = 80 encroaching, for each ecosystem).

To estimate carbon sequestration in intact ecosystems and encroaching areas we used the methodology published by Pearson [20]. During June–July of 2016, we identified locations of CPM, OPB, and WMP within ASGA and MSGA from unpublished information through MNFI. e randomly positioned 10 circular plots (4 m radius) within 2 replicates of each targeted intact ecosystem (CPM, OPB, and WMP) and at paired locations in adjacent encroaching forests, for a total of 40 sites per ecosystem (20 from intact locations and 20 from encroaching, Fig 2). Within each circular plot, we identified each tree (DBH > 5 cm) to genus and measured the diameter at breast height (DBH). Within 2, 0.25 m^2^ subplots, we harvested living herbaceous and dead biomass (primarily leaf litter) and collected 2 soil samples with a soil auger (30.5 cm length, 2 cm diameter) to measure soil organic carbon (SOC).

We determined tree aboveground carbon stock using allometric equations (Table 2) applied to the diameter measurements of hardwood and softwood species groups [20] and used the plot area to estimate the amount of carbon stored in trees on a per-hectare basis. Living herbaceous biomass (i.e., from clipped herbaceous vegetation) and dead organic matter (DOM) samples were dried at 105 C for at least 24 hours and then weighed to determine the aboveground biomass of herbaceous species and DOM per hectare. To convert biomass to metric tonnes of carbon/ha, we multiplied values by 0.5.

**Table 2:** Equations used to estimate tree biomass (kg) from diameter at breast height (cm) from Jenkins et al. [23]. Genera presented include all measured trees in this study.

Genus	Species Group	Equation*
*Acer*	Hard maple/oak	y = exp(-2.0127 + 2.4342 * ln x)
*Ostrya*	Mixed hardwood	y = exp(-2.4800 + 2.4835 * ln x)
*Pinus*	Pine	y = exp(-2.5356 + 2.4349 * ln x)
*Populus*	Aspen/alder/cottonwood/willow	y = exp(-2.2094 + 2.3867 * ln x)
*Quercus*	Hard maple/oak	y = exp(-2.0127 + 2.4342 * ln x)
*Sassafras*	Mixed hardwood	y = exp(-2.4800 + 2.4835 * ln x)

y = total aboveground biomass (kg)

x = diameter at breast height (cm)

To compute SOC, we first quantified bulk density by dividing the weight of dried (105 C for 24 hours) soil samples by the auger volume. Next, we sieved homogenized soil samples with a #45 sieve (355 μm openings) and added approximately 2–5 g of the sample to dry, warm crucibles which were previously weighed to the thousandth of a gram [24]. We determined pre-combustion soil weight to the nearest thousandth of a gram by subtracting the dry crucible weight from the combined weight of the crucible and soil sample. We then burned off soil organic matter in a muffle furnace at 500 C for five hours and weighed samples again to determine percent carbon per sample [24, 25]. To compute metric tonnes of carbon/ha, we used the equation:

$$\text{SOC}\left(\text{t/ha}\right)=[\left(\text{soil}\;\text{bulk}\;\text{density}\left({\text{g/cm}}^{\text{3}}\right)\times \text{soil}\;\text{depth}\left(\text{cm}\right)\times \%\text{carbon}\right]*100$$

### Spatial and statistical analyses

First, we compared mean values of abiotic data from intact ecosystems and encroaching areas. Next, we summed carbon from trees, herbaceous species, dead organic matter, and soil organic carbon, hereafter referred to as “total carbon”. We then used Shapiro-Wilk and Levene’s Test to assess assumptions of normality and equal variance of total carbon in metric tonnes (t/ha) and SOC (t/ha). When necessary, data were log10 transformed to meet assumptions of normality. To compare total carbon and SOC of intact and encroaching ecosystems, we used both a 2-way ANOVA and post-hoc t-tests for multiple comparisons with a Holm’s adjustment, and a principal component analysis (PCA) to compare intact ecosystems and their encroaching areas using all measured components of carbon stocks.

Using leaf-on 2016, 1 m resolution digital ortho photography imagery from the National Agriculture Inventory Program [26], we calculated the spectral reflectance signatures of the red, green, blue, and infrared bands. We used NAIP aerial imagery because it is accessible, high resolution, and commonly used in ecological studies. Using these data, we computed all possible combinations of ratios between pairs of spectral bands. To create predictive models for total carbon and SOC, we used simple linear regression with spectral reflectance, and/or ratios between bands as independent variables. If linear models were significant in predicting total carbon or SOC using the reflectance properties of a multispectral image, we applied models spatially to determine how well images could explain total carbon and SOC in a natural area. To test the validity of the models, we classified 2016 NAIP imagery within ASGA and MSGA study areas into three land classes: forest, non-agricultural openings, and “other” using supervised classification with a maximum likelihood classifier in ArcGIS Pro v 3.1. Agriculture and impervious surfaces were filtered out, as models do not apply to human-altered areas. This land cover classification was necessary to stratify the imagery based on land cover type and create a single surface predicting total carbon and SOC, both using two separate models (i.e., one for the rare intact ecosystem itself, and one for the encroaching forested areas). We ran each SOC model for the intact and encroaching areas associated with each ecosystem type at ASGA using the NAIP imagery and then used the “conditional” tool in Spatial Analyst to combine models into a single surface. The result was a single raster model depicting SOC for each ecosystem type and the encroaching forested areas at ASGA. We then applied the same procedure to known occurrences of these rare ecosystems at MSGA and compared the resulting values of SOC within the rare ecosystems at MSGA to those at ASGA.

We conducted all exploratory data and statistical analyses using R [27]; specifically, the packages vegan [28], UsingR [29], and car [30]. Spatial analyses were conducted using the analysis and spatial analyst toolsets in ArcGIS Pro v3.1.

## Results

Abiotic parameters (humidity, wind speed, air temperature, soil temperature, and soil pH) were similar for each ecosystem, with differences likely a function of soil type and the difference in overstory growth (Tables 3 and 4). The wetland environments, CPM and WMP, were more humid and had cooler soil temperatures than OPB, however, WMP had greater wind speeds. Not surprisingly, the encroaching habitats had more trees with greater mean DBH relative to the intact ecosystems, which generally have decreased canopy cover (Table 3).

**Table 3:** Mean humidity (Humid), wind speed (WindSp), air temperature (AirT), soil temperature (SoilT) and soil pH for intact and areas encroaching intact ecosystems with standard error (n = 20).

Ecosystem	Type	Humid (%)	WindSp (kph)	AirT (C)	SoilT (C)	Soil pH
Coastal Plain Marsh	Intact	71.60 ± 0.83	1.85 ± 0.02	27.30 ± 0.16	20.65 ± 0.50	6.68 ± 0.07
	Encroaching	70.61 ± 2.54	1.74 ± 0.16	27.82 ± 0.27	18.90 ± 0.37	6.98 ± 0.03
Oak-Pine Barrens	Intact	67.86 ± 1.77	1.00 ± 0.23	25.26 ± 0.66	22.65 ± 0.59	7.00 ± 0.00
	Encroaching	65.38 ± 2.24	2.19 ± 0.56	26.50 ± 0.46	20.40 ± 0.64	6.70 ± 0.08
Wet-Mesic Sand Prairie	Intact	71.93 ± 1.75	3.23 ± 0.64	19.99 ± 0.45	19.25 ± 0.68	6.70 ± 0.08
	Encroaching	55.42 ± 1.46	4.22 ± 0.74	19.65 ± 0.45	18.00 ± 0.56	6.98 ± 0.03

**Table 4:** Mean diameter at breast height (cm) with standard error and the number of individuals, sum of total biomass per genus (kg), and total carbon per genus (t) for all trees measured in intact and encroaching areas of rare ecosystems.

Ecosystem	Type	Genus	Mean DBH (cm)	Sum Total Biomass (kg)	Total Carbon (t)
Coastal Plain Marsh	Intact	Quercus	20 (1)	190.4	0.19
	Encroaching	Quercus	17.8 ± 1.55 (59)	16,049.9	16.05
		Acer	8.4 ± 1.06 (8)	227.3	0.23
		Pinus	6.4 (1)	7.3	0.01
		Sassafras	5.7 (1)	6.3	0.01
Oak-Pine Barrens	Intact	Quercus	14.6 ± 5.40 (6)	1,311.7	1.31
		Prunus	15.7 ± 2.83 (5)	487.1	0.49
		Acer	25.2 (1)	344.5	0.34
	Encroaching	Quercus	20.2 ± 1.77 (30)	8,546.1	8.55
		Pinus	10.6 ± 1.58 (9)	293.6	0.29
		Prunus	16.3 ± 0.25 (2)	170.3	0.17
		Acer	11.5 (1)	51.0	0.05
Wet-Mesic Prairie	Intact	Quercus	17.3 ± 2.96 (6)	1,037.0	1.04
		Populus	6.7 ± 0.85 (3)	31.7	0.03
	Encroaching	Quercus	19.3 ± (38)	11,311.0	11.31
		Acer	12.0 ± 2.43 (9)	812.5	0.81
		Pinus	12.4 ± 2.39 (8)	431.1	0.43
		Prunus	14.3 ± 3.95 (3)	238.8	0.24
		Ostrya	11.9 ± 1.3 (3)	123.0	0.12
		Populus	5.8 ± 0.33 (3)	22.4	0.02

**Fig 3:**
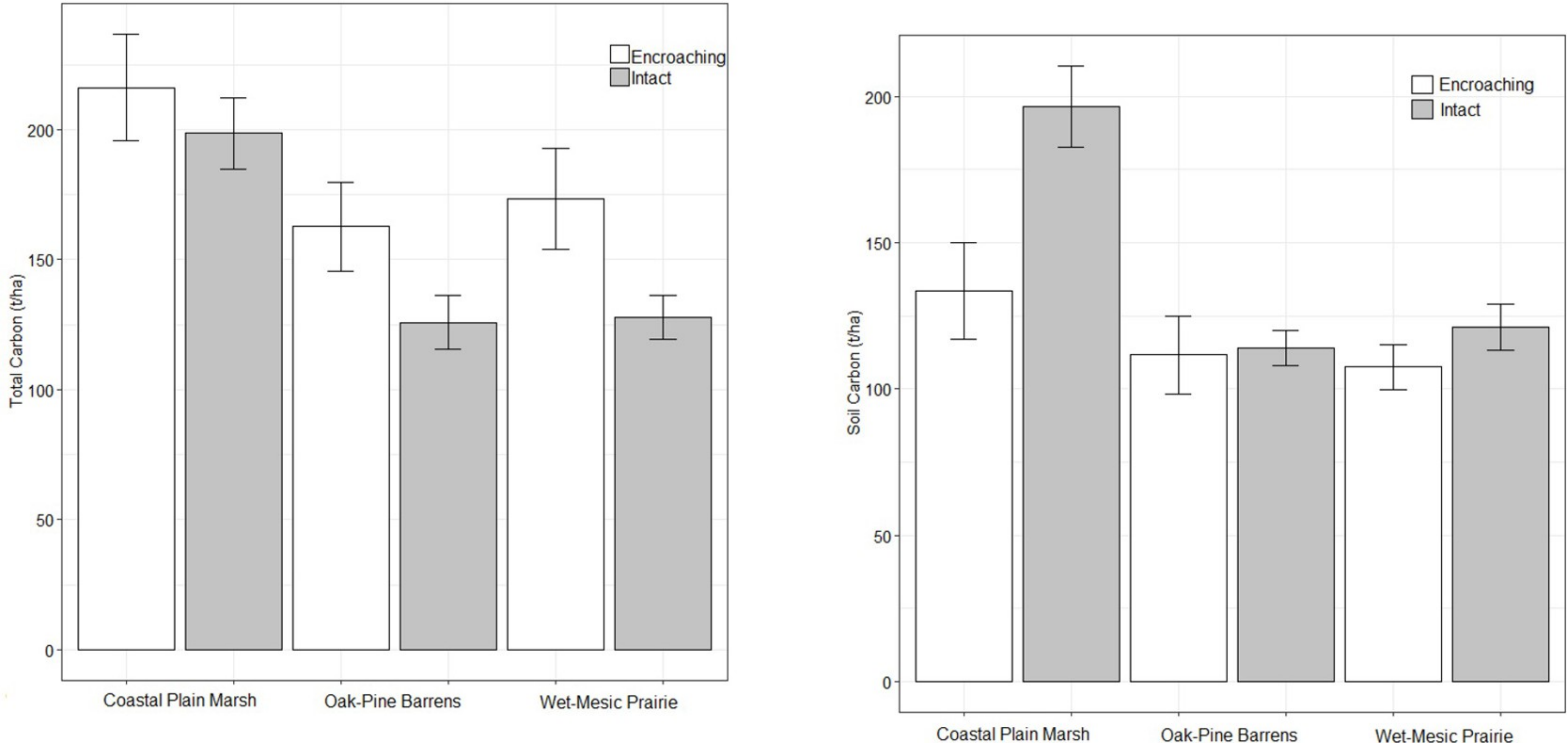
Mean total carbon (A) and soil organic carbon (B) in intact Coastal Plain Marsh, Oak-Pine Barrens, and Wet-Mesic Sand Prairie and their encroaching forests with standard error bars. Total carbon was greater in encroaching forests than intact systems and great in CPM compared to OPB and WMP. Soil organic carbon was greater in intact ecosystems relative to encroaching and in CPM relative to other ecosystems.

We removed 2 outliers (> 2 standard deviations ± mean) from SOC values of encroaching OPB. Total carbon differed by ecosystem (F(_2,112_) = 10.3162, p = 7.724e-05) and as a function of encroachment (F(_1,112_) = 6.3578, p = 0.01309); whereas the interaction of the two, was not significant (F(_2,112_) = 0.5469, p = 0.58029; Table 5 and Fig 3A). Post hoc tests with Holm’s adjustment revealed that CPM had greater total carbon than either OPB (p = 0.00031) or WMP (p = 0.00092) (Fig 3A) and encroaching areas had more total carbon than intact ecosystems (p = 0.01309).

**Table 5:** Mean and standard error for total, tree, herbaceous, dead organic matter (DOM), and soil organic carbon (SOC) in tC/ha, along with bulk density of soil (g/cm3). Intact and encroaching areas of CPM and WMP had 20 samples, whereas OPB had 20 samples in intact and 18 samples in encroaching areas, where 2 outliers were removed.

Ecosystem	Coastal Plain Marsh		Oak-Pine Barrens		Wet-Mesic Prairie	
Type	Intact	Encroaching	Intact	Encroaching	Intact	Encroaching
Total (tC/ha)	198.45 ± 13.73	216.12 ± 20.55	125.82 ± 10.37	162.58 ± 13.19	127.81 ± 8.61	173.35 ± 19.28
Tree (tC/ha)	0.95 ± 0.95	81.01 ± 18.66	10.50 ± 7.58	45.06 ± 14.86	5.31 ± 3.65	64.34 ± 18.48
Herb (tC/ha)	0.22 ± 0.02	0.10 ± 0.03	0.20 ± 0.04	0.09 ± 0.02	0.33 ± 0.04	0.09 ± 0.02
DOM (tC/ha)	0.68 ± 0.10	1.50 ± 0.09	1.04 ± 0.13	1.59 ± 0.17	1.11 ± 0.08	1.51 ± 0.11
SOC (tC/ha)	196.60 ± 13.82	133.52 ± 16.53	114.09 ± 6.03	111.58 ± 17.12	121.06 ± 7.90	107.42 ± 7.62
BD (g/cm3)	1.17 ± 0.02	1.03 ± 0.02	1.20 ± 0.02	1.07 ± 0.01	1.09 ± 0.02	1.08 ± 0.02

Although SOC differed by system (F(2,112) = 7.6290, p = 0.0007831) and degree of encroachment (F(1,112) = 9.4579, p = 0.0026420), these differences were driven by high SOC in CPM (Fig 3B). The interactive term was not significant (F(2,112) = 2.6236, p = 0.0769999).

**Fig 4:**
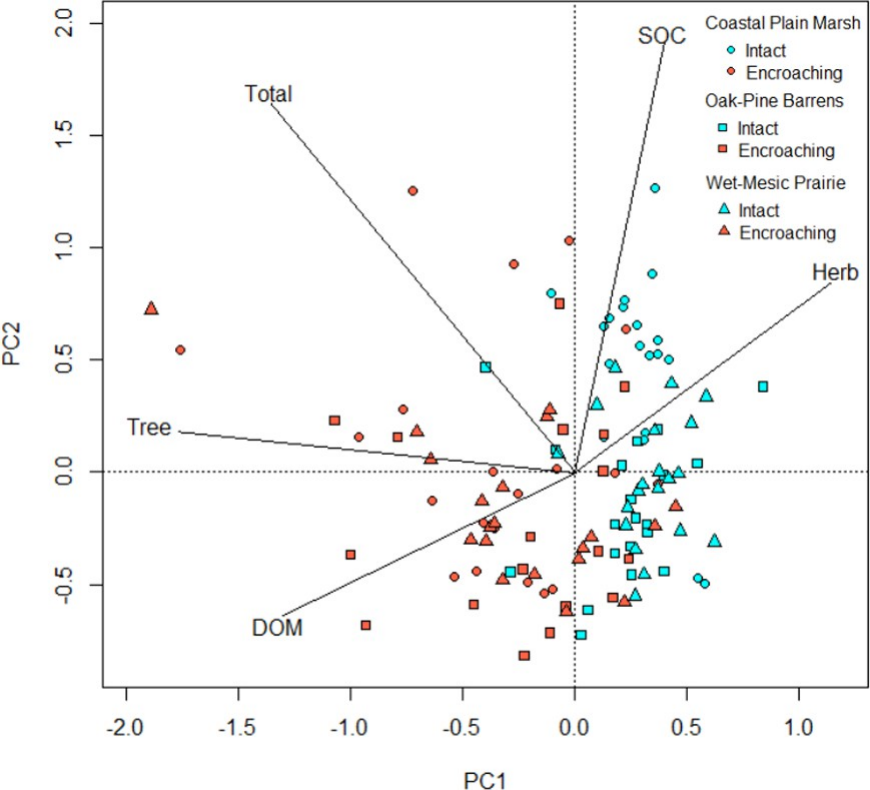
Principal component analysis based on the correlation matrix of standardized parameters (SOC–soil organic carbon, herb–herbaceous, DOM–dead organic matter, tree–aboveground trees, and total–sum of carbon stocks in all measured components); variation explained in PC1 and PC2 was 72%.

A two-dimensional ordination following PCA of the correlation matrix explained 72% of the variation in the dataset (Fig 4). This included carbon stocks in trees, dead organic matter, herbaceous species, SOC, and total carbon for intact and encroaching ecosystems. Each of the five descriptors had a similar magnitude of eigenvector in the ordination. The wetlands (CPM and WMP) had greater SOC and herbaceous carbon, relative to their encroaching areas which had greater carbon stocks in trees and DOM (Fig 4).

Before linear modeling, we removed two outliers (>2 standard deviations ± mean) from intact CPM. Models that were not significant were not included in spatial analyses. Total carbon stocks and SOC in intact CPM and WMP were significantly explained by the ratio of reflected red and green light (Tables 6 and 7). We assessed models using root mean square error (Tables 6 and 7) and validated significant models using images from MSGA.

**Table 6:** Linear model describing total carbon (includes carbon stocks in trees, herbaceous plants, dead organic matter, and SOC) in intact coastal plain marsh and wet-mesic prairie based on the ratio of reflected red to green light; forests encroaching wet-mesic sand prairie explained by the ratio of green to blue light. Note: models for oak-pine barrens were not significant and are thus not included in the table.

Ecosystem		Coefficient	p-value_coef_	R^2^	p-value_model_	RMSE
Intact	R:G	388.42	0.00595	0.3857	0.005945	29.72007
Coastal Plain Marsh	intercept	-84.28	0.38579			
Intact	R:G	-214.68	0.010659	0.3108	0.01066	31.14771
Wet-Mesic Sand Prairie	intercept	306.8	1.29E-04			

**Table 7:** Linear model describing soil organic carbon in intact coastal plain marsh and wet-mesic sand prairie based on the ratio of reflected red to green light. Note: models for oak-pine barrens were not significant and are thus not included in the table.

Ecosystem		Coefficient	p-value_coef_	R^2^	p-value_model_	RMSE
Intact	R:G	382.04	0.01	0.3477	0.01001	31.72848
Coastal Plain Marsh	intercept	-81.39	0.432			
Intact	R:G	-218.57	0.00365	0.3825	0.003652	27.05687
Wet-Mesic Sand Prairie	intercept	303.3	3.06E-05			

**Fig 5:**
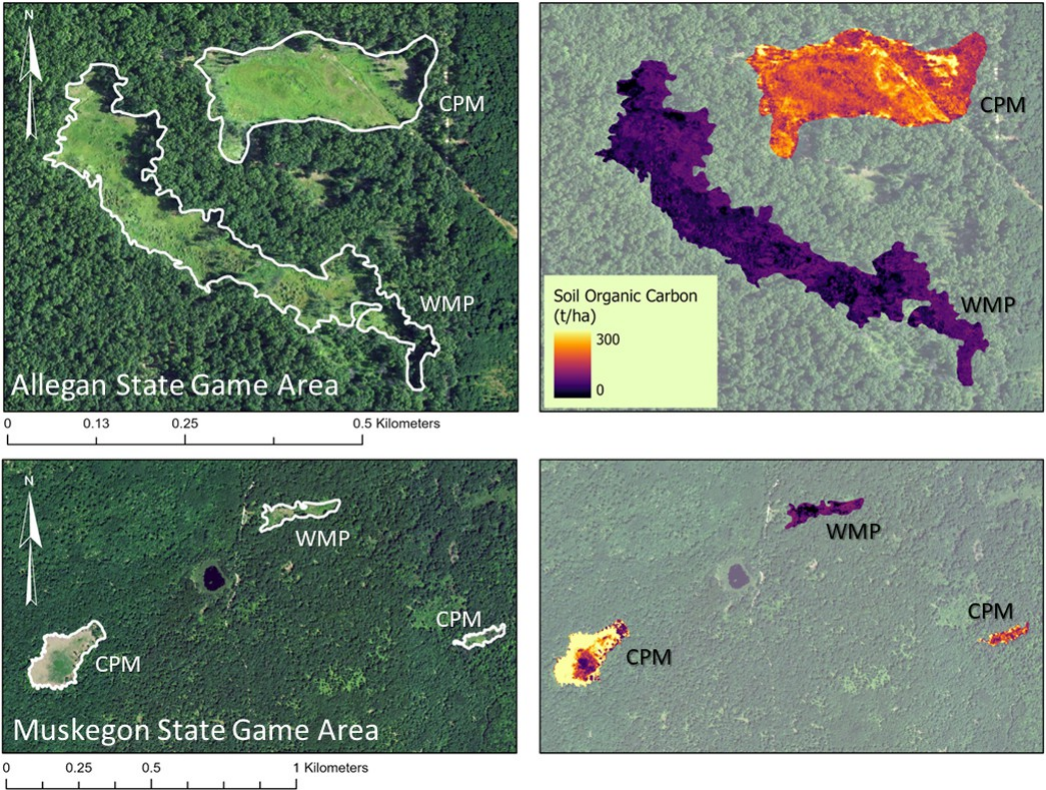
Map of soil organic carbon (SOC) within 2 rare ecosystems (Coastal plain marsh–CPM and wet-mesic sand prairie–WMP) in Allegan State Game Area (ASGA) compared to predicted values of similar ecosystems at Muskegon State Game Area (MSGA). The model was created using soil data collected from ASGA and computed SOC from reflectance in 4-band 1 m resolution imagery (acquired from the National Agriculture Inventory Program [26]). The model was validated by applying to known CPM and WMP ecosystems within MSGA. Predicted SOC values were similar between ASGA and MSGA.

Spatial application of the models over the extent of CPM and WMP at ASGA revealed that predicted values for SOC approximated SOC using field measurements (Table 5 and Fig 5). Further, model validation revealed that predicted SOC values at MSGA were like those at ASGA for CPM and WMP (Fig 5). Values for SOC at one of the CPMs at MSGA (southwest corner, Fig 5) were greater than those for ASGA due to patchiness of vegetation at that CPM site in comparison to the others. Sparse vegetation in these patchy areas yielded high ratios of R:B, suggesting model performance decreases with less vegetation.

## Discussion

We quantified carbon in soil (SOC), dead organic matter (DOM), the herbaceous layer, and above and belowground tree biomass in three rare ecosystems (oak-pine barrens, coastal plain marsh, and wet-mesic sand prairie) in western Lower Michigan. We found that total carbon was greater in the encroaching areas relative to intact OPB, CPM, and WMP and SOC was greater in intact relative to encroaching areas, driven by high SOC in CPM. Our PCA results suggest that when considering DOM, herbaceous species, SOC, and aboveground trees, these rare systems sequester carbon differently than encroaching forests (Fig 4). The interior of CPM and WMP had greater carbon stocks in the soil and herbaceous biomass, while encroaching areas of these ecosystems had greater carbon stocks in tree biomass and DOM. These patterns likely reflect the vegetation and hydrologic characteristics in the interior and encroaching areas of these ecosystems [6, 31].

Oak-pine barrens and similar ecosystems such as oak savannas and deciduous forests have been shown to sequester as much as 0.4 Pg C/year in soils and 1–3 Pg C/year total globally (Lal, 2005). Unit estimates of carbon storage in these ecosystems (e.g., oak barrens, oak savannas, oak-pine forests) vary depending on location. For instance, Puhlick and Weiskittel [32] inventoried all ecological reserves in Maine and determined that on average oak-scrub barrens stored around 56 tC/ha in aboveground biomass (i.e., aboveground living biomass, dead wood, and litter) and over 100 tC/ha in soils, while Smith and Heath [33] determined that carbon density in all north eastern U.S. forests was estimated to be over 600 tC/ha in soil and over 200 tC/ha for all other carbon stocks (i.e., above and belowground, dead wood, and litter). In a study by Heise [34], SOC and total carbon (above and belowground tree, dead wood, and litter) in oak savannas in southwest Michigan was estimated to be between 15–25 tC/ha and over 20,000 tC/ha, respectively, depending on restoration stage and degree of encroachment. We measured values for SOC in OPB like those that Puhlick and Weiskittel [32] reported, and values of total carbon more similar to those that Smith and Heath [33] reported. Areas encroaching on intact OPB contained greater total carbon, but no difference in SOC (Table 3 and Fig 3).

Coastal plain marshes, along with other wetland ecosystems, are known to store vast amounts of carbon in their soils [6]. As part of a large study where 967 wetland points in the conterminous United States were sampled for SOC stock and density, Nahlik and Fennessy [6] estimate that freshwater wetlands in the conterminous U.S. stored an average of 539 tC/ha in the top 100 cm of soil, and between 15 and 109 tC/ha in the top 30 cm of soil. Braun et al. [13] reports SOC from the top 20–30 cm of soil in freshwater marshes near Illinois Beach to range 100–240 tC/ha. The values we report from the top 30 cm of soil in both intact (mean ± standard error: 196.596 ± 13.818 tC/ha) and encroaching areas (mean ± SE: 133.518 ± 16.525 tC/ha) of CPM are comparable to those reported by Nahlik and Fennessy [6] but are closer to estimates reported by Braun et al. [13]. The values we report for total carbon are much higher than those found in some studies [35, 36], but comparable to those found in other studies [37]. We found no difference in total carbon between intact and encroaching areas of CPM, but higher SOC in the intact than in the encroaching areas.

Carbon stored in soil is more recalcitrant and secure long term than carbon stored in aboveground biomass, which can be released more easily due to disturbances [38]. Some studies have found similar relationships between soil carbon in intact and encroaching areas [31], while others report no net change in soil carbon [39–41], or a loss in soil carbon [42] upon encroachment. The dynamics of soil carbon storage in encroached ecosystems can depend on hydrological characteristics [6], which is likely the main reason we see more soil carbon in intact CPM than in their encroaching areas. Wetlands tend to store more soil carbon than other ecosystems due to their anoxic conditions that slow decomposition and lead to the accumulation of organic matter [6].

In addition, the dynamics of soil carbon storage in encroached ecosystems can depend on the vegetation community [31], annual precipitation [42], soil properties [43, 44], and/or the length of time the ecosystem has been encroached upon [45]. For example, McCulley and Jackson [31] found that grasses allocate proportionally more carbon belowground than woody species when comparing an intact tallgrass prairie to one encroached upon by woody vegetation in central Texas. Asner et al. [43] determined that clay loam soils supported substantially larger carbon pools than shallow clay soils across a 400km² area of northern Texas that had a 32% increase in aboveground woody plant carbon pools over 63 years. Throop and Lajtha [45] found that surface soil carbon increased over time upon the encroachment of juniper into sagebrush steppe in the western US due to increases in the size of the juniper plants. Although these studies were not conducted in the Great Lakes region, we still would expect soil carbon dynamics in our system to vary with these and potentially other factors. Our study does not incorporate a temporal component, which would help better determine the long-term impacts of these rare ecosystems on carbon sequestration in western Lower Michigan. Furthermore, our study does not incorporate soil properties or other factors that may be influencing soil carbon levels, which should be further studied.

Wet-mesic sand prairies, like other types of prairie (e.g., tallgrass), can store large amounts of carbon (~0.5 Mg C/ha/year; [46]) especially when compared to land uses that often replace them (e.g., agriculture, urban; [47, 48]). However, studies have shown that encroaching areas of prairie and grassland ecosystems can have higher levels of total carbon than soil carbon due to high aboveground primary production and biomass [41, 49]. Since encroaching areas were forested, it is not surprising that they contained a greater number of trees (WMP: all encroaching sites = 64 trees, all intact sites = 9 trees; CPM: all encroaching sites = 72 trees, all intact sites = 1 rotten tree, Table 2) and were dominated by maple and oak species, which often store more carbon aboveground as compared to wetlands and grasslands.

Total carbon was significantly higher in the encroaching areas than in the interior of OPB and WMP and this was driven by aboveground tree carbon. This is reflected in our PCA results as well, which show that carbon stocks are mainly in trees. Field et al. [50] described the phenomenon of “woody encroachment” as the conversion of grasslands to savannas and savannas to shrublands. This encroachment, although seemingly disruptive to the original ecosystem, might present an increase to the aboveground carbon sink [50]. Others have also found that the encroachment of woody vegetation may lead to increased aboveground carbon storage [41, 51] and ultimately contribute to carbon sequestration [52, 53]. For example, Hughes et al. [41] determined that mesquite encroachment into savanna ecosystems in Texas led to increases in total aboveground carbon depending on soil type. Similarly, Lett et al. [51] found that *Cornus drummondii* encroachment into mesic grasslands of the central U.S. also led to increased aboveground carbon storage.

When applied to multispectral imagery, the SOC predictions held consistent between the ASGA study area and the MSGA validation site. The image reflectance in the wetlands consistently predicted SOC values within the range of SOC as measured from field data (Table 3). Shadows or other small openings in the forest canopy were problematic when presenting the model spatially, as the reflectance in shadowed areas is indicative neither of forest canopy nor wetland openings. Thus, speckled, or spotty patterns of high SOC are displayed in the predictive maps where shadows or patchy vegetation are evident in the imagery (Fig 5). These areas are inevitable in high-resolution imagery. Similarly, there are areas inside the wetlands that have small patches of trees and are interpreted as forest cover in the land cover classification. As such, when the wetland SOC algorithm was applied to those areas, SOC estimates were lower than our measurements despite occurring within a wetland. While the trees within the wetland systems likely do have an impact on SOC estimates in their immediate vicinity, the actual SOC of the soil may be more like that of the wetland vegetation reflectance. Regardless, the pattern of SOC predictions yields areas that are distinctly different between wetlands and encroaching forest areas. When applied across a landscape, these predictions may identify carbon sinks that may be important conservation considerations. The models for OPB likely were not significant in our analysis because the overstory tree canopy was much higher than in CPM and WMP. Thus, reflection values from the imagery may have been more representative of canopy and/or shadows from trees rather than the soil properties at the sampling location. Other variables such as brightness of infrared, red, or green bands in the imagery or vegetation and temperature condition index may need to be used in addition to NDVI for reliable prediction of soil carbon [54].

## Conclusions

Encroaching areas threaten intact rare ecosystems with conversion to greater tree density and basal area of forest vegetation likely resulting in dryer soils (relative to CPM and WMP) and fewer barrens (relative to OPB). In rare wetland ecosystems, this change to greater aboveground biomass shifts carbon from soils to trees. Our models provide an accurate and accessible estimation of carbon stored in the soil of these rare ecosystems, which may provide a useful tool in their conservation. Conserving these rare ecosystems will allow for continued carbon sequestration, which may aid in slowing global warming and climate change.
